# Fatal Case of Lassa Fever, Bangolo District, Côte d’Ivoire, 2015

**DOI:** 10.3201/eid2509.190239

**Published:** 2019-09

**Authors:** Mathieu Mateo, Caroline Picard, Yahaya Sylla, Emilie Kamo, Danielle Odegue, Alexandra Journeaux, Stéphane Kouassi Kan, Marcelle Money, David N’Golo Coulibaly, Eugène Koffi, Souleymane Meite, Véronique Akran, Hervé Kadjo, Edgard Adjogoua, Solange N’Gazoa Kakou, Sylvain Baize, Mireille Dosso

**Affiliations:** Institut Pasteur, Lyon, France (M. Mateo, C. Picard, A. Journeaux, S. Baize); Centre International de Recherche en Infectiologie, Lyon (M. Mateo, C. Picard, A. Journeaux, S. Baize);; Institut Pasteur de Côte d’Ivoire, Abidjan, Côte d'Ivoire (Y. Sylla, E. Kamo, D. Odegue, S.K. Kan, M. Money, D. N’Golo Coulibaly, E. Koffi, S. Meite, V. Akran, H. Kadjo, E. Adjogoua, S. N’Gazoa Kakou, S. Baize, M. Dosso)

**Keywords:** Lassa fever, Lassa fever virus, viruses, retrospective identification, fatal case, zoonoses, Bangolo District, Côte d’Ivoire

## Abstract

Lassa fever has not been reported in Côte d’Ivoire. We performed a retrospective analysis of human serum samples collected in Côte d’Ivoire in the dry seasons (January–April) during 2015–2018. We identified a fatal human case of Lassa fever in the Bangolo District of western Côte d’Ivoire during 2015.

Lassa fever is endemic to western Africa. Nigeria, Guinea, Sierra Leone, and Liberia regularly have outbreaks of Lassa fever, mostly during the first few months of the year, corresponding to the dry season (January–May), when the *Mastomys natalensis* rodent reservoir of Lassa fever virus (LASV) has more contact with the human population in rural areas to access food. The epidemic zone of Lassa fever has recently been extended into Benin, and sporadic cases have been documented in Burkina Faso, Mali, Ghana, and Togo.

Côte d’Ivoire appears to be an exception; no Lassa fever cases have been reported in this country. A tourist from Germany traveling through Côte d’Ivoire, Burkina, and Ghana died from Lassa fever upon her return to Germany but it was not possible to determine in which country she contracted the disease (*1*). In 2013, LASV RNA was identified in *M. natalensis* rodents captured in northern Côte d’Ivoire, near Korhogo (*2*). Virus RNA corresponded to the same AV strain of LASV as that isolated from the tourist, suggesting that she might have been infected in Côte d’Ivoire. Seroprevalence among forest workers in western Côte d’Ivoire also suggests that LASV might currently circulate in this country (*3*).

We performed a retrospective analysis of 268 human serum samples received at the National Reference Center for Yellow Fever (Institut Pasteur de Côte d’Ivoire, Abidjan, Côte d’Ivoire) for diagnosis of arbovirus infection. We selected yellow fever–negative samples from the western region of Côte d’Ivoire (Biankouma, Danané, Duékoué, Guiglo, Man, Odienné, Touba, Toulépleu, and Zouan Hounien Districts), near the borders with Liberia and Guinea collected during January–April 2015–2018.

We inactivated serum samples by using AVL buffer (QIAGEN, https://www.qiagen.com) and ethanol and isolated RNA by using the QIAamp Viral RNA Extraction Kit (QIAGEN). We analyzed RNA by using a reverse transcription PCR (RT-PCR) and pan–Old World arenavirus primers specific for the large RNA segment (OW RT-PCR).

We identified 1 positive serum sample (001/15) by OW RT-PCR; we then determined that this sample was LASV positive by using a LASV-specific RT-PCR specific for the glycoprotein complex (GPC) gene (Figure, panel A). We sequenced amplicons for the GPC and L genes and aligned partial sequences of this new strain, Bangolo-CIV-2015, with the corresponding regions of a set of representative published LASV strains (*4*).

**Figure:**
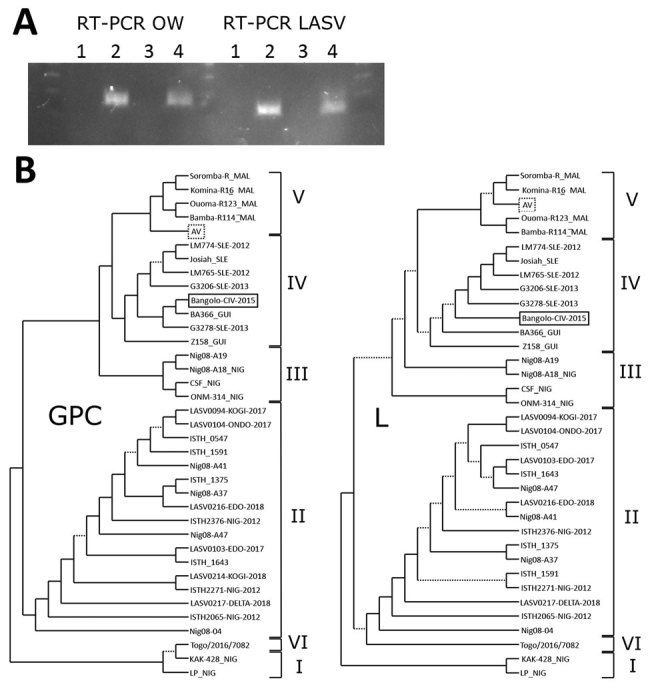
Analysis of LASV strains, Bangolo District, Côte d’Ivoire, 2015. A) RT-PCR analysis of human serum samples 132/16 and 001/15 by using OW and LASV RT-PCRs. Lane 1, negative control; lane 2, positive control; lane 3, 132/16; lane 4, 001/15. B) Phylogenetic analysis of LASV strains. Trees were inferred by using the PhyML Smart Model Selection (*5*) general time-reversible plus gamma plus proportion of invariable sites model with 200 bootstrap replicates. Poorly supported branches with bootstrap values <0.50 are indicated by dotted lines. Lassa virus lineages are indicated by the Roman numerals on the right. The Bangolo-CIV-2015 strain (solid box), which was isolated in this study, appears to be related to clade IV, and the AV strain (dotted box) is related to clade V. GPC, glycoprotein complex gene; L, large RNA segment; LASV, Lassa virus; OW, Old World; RT-PCR, reverse transcription PCR.

We generated phylogenetic trees by using a general time reversible plus gamma plus proportion of invariable sites model and parallel maximum-likelihood with PhyML Smart Model Selection (*5*). The GPC (MK978784) and large RNA (MK978785) fragments of the Bangolo-CIV-2015 strain (Figure, panel B) were genetically similar to strain BA-366 isolated from an *M. natalensis* rodent captured in the Bantou District of central Guinea during 2003 (*6*). On the basis of these phylogenetic trees, we concluded that Bangolo-CIV-2015 belongs to the IV clade, along with the highly pathogenic LASV strain Josiah, and diverges from the clade V AV strain of LASV (Figure, panel B) found in rodents and a patient from Germany (*1*,*2*).

The LASV-positive serum sample originated from a 30-year-old man from the Bangolo District of Côte d’Ivoire who was admitted to Duékoué Hospital in January 2015 because of fever, asthenia, and gingivorrhagia. His health rapidly deteriorated after admission; he had hypotension and a consciousness disorder and died 4 days later. The sample was collected at the time of death. Further investigations by doctors at Institut Pasteur de Côte d’Ivoire were unable to obtain more information about this patient. Without the travel history of the patient during the 3 weeks preceding his hospital admission, we could not determine whether this case of Lassa fever was endemic or imported. Exported cases are common (*7*) because many workers travel to Côte d’Ivoire from Guinea and Sierra Leone.

Next-generation sequencing in an outbreak setting, combined with phylogenetic analyses, has recently showed that many strains of LASV have been responsible for cases of Lassa fever in Nigeria, suggesting independent transmission events from the reservoir, rather than the emergence of an epidemic strain (*8*). The case we report remains isolated because no other suspected cases were reported during this period. None of the healthcare workers who had been in contact with the patient showed any signs of Lassa fever. An additional set of 35 human serum samples collected during October 2014–April 2015 in the Biankouma, Duékoué, Guiglo, Issia, Man, Minignan, Odienne, Soubre, and Tengrela Districts were LASV negative by RT-PCR.

In 2015, Kouadio et al. highlighted possible underreporting of Lassa fever cases in Côte d’Ivoire because of lack of diagnoses (*2*). We provide evidence of a fatal case of human Lassa fever in the Bangolo District of western Côte d’Ivoire. Thus, measures should be taken to reinforce the diagnosis of Lassa fever and arenavirus surveillance in general in this country. Human serologic surveys should help in identifying the area of LASV circulation in Côte d’Ivoire. RNA from novel arenaviruses has recently been identified in rodents captured in Côte d’Ivoire, but their pathogenic potential for humans remains unknown (*9*).
